# A simple electrochemical immunosensor based on a chitosan/reduced graphene oxide nanocomposite for sensitive detection of biomarkers of malignant melanoma†

**DOI:** 10.1039/d2ra04208h

**Published:** 2022-09-12

**Authors:** Huihua Zhang, Hui Qu, Jingbo Cui, Linxia Duan

**Affiliations:** Shanxi Bethune Hospital, Shanxi Academy of Medical Sciences, Tongji Shanxi Hospital, Third Hospital of Shanxi Medical University Taiyuan 030032 China zhanghh_hust@126.com; Tongji Hospital, Tongji Medical College, Huazhong University of Science and Technology Wuhan 430030 China

## Abstract

The sensitive and specific detection of tumor biomarkers is crucial for early diagnosis and treatment of malignant melanoma. Immunoassay with a simple sensing interface and high sensitivity is highly desirable. In this work, a simple electrochemical immunosensor based on a chitosan/reduced graphene oxide (CS–rGO) nanocomposite was developed for sensitive determination of an S-100B protein, a tumor marker of malignant melanoma. CS–rGO nanocomposite were prepared by chemical reduction of graphene oxide in the presence of chitosan and modified on glassy carbon electrode (GCE) to provide a biofriendly, conductive, and easily chemically modified matrix for further immobilization of antibodies. Anti-S-100B antibodies were grafted onto the chitosan molecules to fabricate the immunorecognition interface by a simple glutaraldehyde cross-linking method. Electrochemical determination of S-100B was achieved by measuring the decreased current signal of solution phase electrochemical probes, which originated from the increased steric hindrance and insulation caused by the formation of antigen–antibody complexes at the electrode interface. Due to the good conductivity, high surface area, excellent biocompatibility, and good film-forming ability of CS–rGO, the constructed immunosensor exhibited good stability, high selectivity and sensitivity, a wide dynamic range from 10 fg mL^−1^ to 1 ng mL^−1^ and a low limit of detection of 1.9 pg mL^−1^ (S/N = 3). Moreover, the sensor was also applicable for the sensitive detection of S-100B protein in real human serum samples.

## Abstract 2

Simple electrochemical immunosensor is easily fabricated based on chitosan/reduce graphene oxide nanocomposite for sensitive determination of a tumor marker of malignant melanoma.

## Graphical abstract

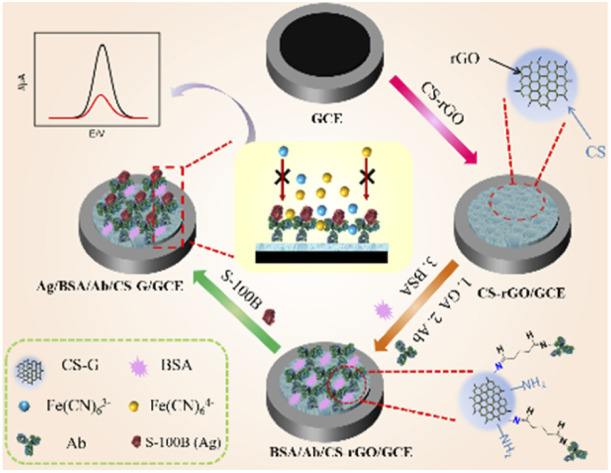

## 1. Introduction

Malignant melanoma is one of the fastest growing malignant tumors.^1^ As the most malignant skin tumor, it can occur in different tissues such as skin, mucous membranes (*e.g.* alimentary tract, respiratory tract, and genitourinary tract),^2^ eye glucose membrane, soft tissue, *etc.*^3^ Malignant melanoma has a lower age of death than other solid tumors.^4^ In addition, malignant melanoma lacks specific treatment other than early surgical resection and has a poor prognosis. Thus, early diagnosis and treatment of malignant melanoma is extremely important to improve survival.^5^ As an acidic calcium-binding protein, S-100B is the first marker to specifically label tumor cells of malignant melanoma and its value in the early diagnosis of malignant melanoma has been fully confirmed.^6^ When the level of S-100B in serum exceeds 0.5 ng mL^−1^, it is generally considered to be a pathological concentration. The concentration range of 0.15–0.5 ng mL^−1^ is between pathological and normal, and the concentration below 0.15 ng mL^−1^ is normal.^7^ Therefore, sensitive detection of S-100B in serum based on a simple method is of great significance.

Until now, quantification of S-100B levels in serum is mainly based on various immunoassay methods including enzyme-linked immunosorbent assay, fluorescence immunoassay, radioimmunoassay, and electrochemiluminescence immunoassay.^8–11^ However, these methods typically suffer from expensive instruments, specialized operating techniques, high costs, or low sensitivity. In contrast, electrochemical immunoassays combine the advantages of rapid detection, simple instrument, low cost, easy integration, and potential for high throughput.^12–14^ It is highly desirable to construct an efficient immunosensor for fast, sensitive, and low-cost detection of S-100B.

The introduction of functional nanomaterials to improve the performance of electro-chemical immunosensors has attracted much attention in recent years.^15,16^ Graphene based materials have recently attracted great interests due to the unique structure, multidimensional scale (*e.g.* zero dimension-0D graphene quantum dots,^17–19^ two dimensional-2D graphene nanosheets,^20–22^ three-dimensional-3D graphene foam^23–25^) and rich properties, such as good electrical conductivity, high surface area, and good hybridization ability with other functional materials *etc.* Thus, graphene based materials have shown great potential in the fields of (bio)sensor, energy storage, and drug delivery, *etc.*^26–31^ However, the graphene material is prone to agglomeration due to the strong van der Waals forces between the layers. It has been proven that biofunctionalization of graphene material can effectively avoid the agglomeration between layers, and improve its hydrophilicity and biocompatibility.^32^ Chitosan (poly(1,4)-2-amino-2-deoxy-β-d-glucan, CS) is a biological polysaccharide obtained by partial deacetylation of natural chitin. As an important biopolymer, CS is rich in sources and has good biocompatibility. The abundant hydroxyl and amino groups in its molecule make it easy to be derivatized and functionalized. Although CS has good film-forming properties, excellent biocompatibility and high chemical reactivity, CS film has poor mechanical property and usually exhibits easy hydrolysis/swelling in water. It has been proven that graphene materials can effectively improve the strength, stability, water resistance of polymer.^33^

Here, a simple electrochemical immunosensor was developed based on CS/reduced graphene oxide (CS–rGO) nanocomposite for sensitive detection of the biomarker of malignant melanoma, S-100B. The reduction of graphene oxide (GO) in presence of CS avoided the agglomerate of graphene materials and the obtained CS–rGO nanocomposite exhibited good dispersibility in water and monolayer structure. When CS–rGO was drop-coated on glassy carbon electrode (GCE), the biocompatible surface with high stability and excellent electron transfer was beneficial for covalent immobilization of recognition antibodies. Sensitive electrochemical detection of S-100B could be realized by utilizing the reduction of the signal of the redox probe (Fe[CN]_6_^3−/4−^) after the formation of the antigen–antibody complex. The sensor construction was simple and easy operated. The introduction of rGO not only improved the electron transfer rate, but also significantly enhanced the stability of the modified film. Since CS–rGO can provide a biocompatible environment for the immobilized antibodies, the fabricated immunosensor exhibited high stability and achieved sensitive electrochemical detection of S-100B in human serum. This work demonstrates an efficient strategy for easy construction of electrochemical immunosensors with simple fabrication, high sensitivity, good stability, providing a new strategy for electrochemical detection of tumor markers.

## 2. Experimental

### 2.1 Materials and reagents

Mouse anti-human S-100B monoclonal antibody (Ab), S-100B antigen, carcinoembryonic antigen (CEA), prostate specific antigen (PSA) and carcinoma antigen 125 (CA125) and carcinoma antigen 199 (CA199) were purchased from Beijing KEY-BIO Biotech Co., Ltd. (Beijing, China). Bone gamma-carboxyglutamate protein (BGP) was purchased from NanJing OkayBio Co., Ltd. (Nanjing, China). Bovine serum albumin (BSA), sodium phosphate dibasic dodecahydrate (Na_2_HPO_4_·12H_2_O), potassium ferricyanide (K_3_[Fe(CN)_6_]), tetrapotassium hexacyanoferrate trihydrate (K_4_[Fe(CN)_6_]) were obtained from Aladdin Biochemical Technology Co., Ltd (Shanghai, China). Sodium dihydrogen phosphate dehydrate (NaH_2_PO_4_·2H_2_O) was obtained from Shanghai Macklin Biochemical Technology Co., Ltd. (Shanghai, China). Phosphate buffer solution (PBS) was prepared using Na_2_HPO_4_ and NaH_2_PO_4_. Human blood serum (healthy man) was provided by Shanxi Bethune Hospital (Taiyuan, China). All other chemicals were of analytical grade and used without further treatment. Ultrapure water (18.2 MΩ cm) was prepared by the Milli-Q system and used throughout the work.

### 2.2 Characterizations

Scanning electron microscopy (SEM) investigation was performed on a SU8100 micro-scope (Hitachi, Japan). The applied acceleration voltage was 10 kV. UV-visible absorption spectroscopy (UV-vis) was obtained by UV-2450 spectrophotometer (Shimadzu, Japan). Transmission electron microscope (TEM) measurement was performed at an acceleration voltage of 200 kV (JEM-2100, JEOL, Japan). Fourier transform infrared spectroscopy (FT-IR) was measured by a Vertex 70 spectrometer (Bruker, Germany) at room temperature on power-pressed KBr pellets. X-ray photoelectron spectroscopy (XPS) analysis was carried out using Mg Kα radiation (250 W, 14 kV) on a PHI5300 electron spectrometer (PE Ltd, USA). Atomic force microscopic (AFM) image was obtained on a Bruker Multimode 8 (Bruker. Inc, USA). All electrochemical experiments included cyclic voltammetry (CV) and differential pulse voltammetry (DPV) were performed on the electrochemical workstation (Autolab PGSTAT302N, Metrohm, Switzerland) using the traditional three electrode system. Briefly, the working electrode adopted bare or modified glassy carbon electrode (GCE). The reference electrode was a silver/silver chloride (Ag/AgCl) electrode saturated with potassium chloride, and the counter electrode was a platinum wire electrode. Raman spectra was measured using a 960FT-Raman spectrometer (Thermo Nicolet, USA).

### 2.3 Preparation of CS–rGO nanocomposite

Graphene oxide (GO) was prepared by Hummers' method. CS–rGO was prepared as previously reported.^34^ Briefly, GO solution (1 mg mL^−1^, 4 mL) was added into CS solution (0.25%, pH = 3.0) with stirring until the solution became clear and transparent. Subsequently, the solution was then sonicated for 30 min before adding hydrazine hydrate (20 μL). Afterwards, the mixture reacted at 80 °C for 3 h with stirring to reduce GO. Then, the product solution was centrifuged at 3000 rpm for 10 min to remove large particles. The supernatant was collected and further centrifuged at 15 000 rpm for 10 min to obtain CS–rGO nanocomposite. The resulting solid was washed thoroughly with 0.1 mM HCl and then dried by lyophilization. Then, CS–rGO power was obtained for the fabrication of immunosensor based on CS–rGO and the electrochemical determination of S-100B.

### 2.4 Fabrication of immunosensor

Glassy carbon electrode (GCE, 3 mm in diameter) was used as the supporting electrode for constructing the immunosensor. Before use, GCE was sequentially polished with 0.3 and 0.05 μm Al_2_O_3_ power slurry to obtain a mirror-like surface. Then, the electrode was thoroughly cleaned with acetone and ultrapure water for 3 min under ultrasonic treatment. The construction of the immunosensor included three steps. Firstly, GCE was modified with CS–rGO nanocomposites. Briefly, 10 μL of CS–rGO solution (0.1 mg mL^−1^) was drop-coated on GCE followed with drying at room temperature. Secondly, S-100B antibody (Ab) was covalently immobilized on CS–rGO/GCE using glutaraldehyde (GA) as the linker. To activate the amino groups in CS molecules, 20 μL GA (0.01 wt%) was drop-coated on the surface of CS–rGO/GCE and incubated at 25 °C for 15 min in dark. After thorough washing with ultrapure water to remove residual GA, the resulting electrode was immersed in the Ab solution (50 μg mL^−1^ in 0.01 M PBS, pH = 7.4). After incubating for 90 min at 25 °C, the unbound Ab on electrode surface was adequately rinsed with PBS (0.01 M PBS, pH = 7.4). Finally, the non-specific sites on the obtained Ab/CS–rGO/GCE were blocked by incubation with BSA (1%) at room temperature for 1 h. After carefully rinsed with PBS (0.01 M, pH = 7.4) to remove residual BSA, the immunosensor was finally obtained and denoted as BSA/Ab/CS–rGO/GCE.

### 2.5 Fabrication of immunosensor

Fe(CN)_6_^3−^/Fe(CN)_6_^4−^ was used as the electrochemical probe to detect S-100B. When Ab on the surface of the immunoelectrode selectively recognized S-100B, the formation of antigen–antibody complex reduced the signal of the electrochemical probe. 0.1 M KCl containing 2.5 mM Fe(CN)_6_^3−^/Fe(CN)_6_^4−^ was used as the electrolyte for the detection of S-100B. The DPV curves before and after incubation the immunosensors with different concentrations of S-100B at 37 °C for 30 min were recorded. For real sample analysis, different concentrations of S-100B were added to fresh human serum to simulate the different levels of S-100B in cancer patients. Then, the obtained serum was diluted by a factor of 50 and S-100B was detected using the immunosensor for the fabrication of immunosensor based on CS–rGO and the electrochemical determination of S-100B.

## 3. Results and discussion

### 3.1 Preparation and characterization of CS–rGO nanocomposite

**Fig. 1:**
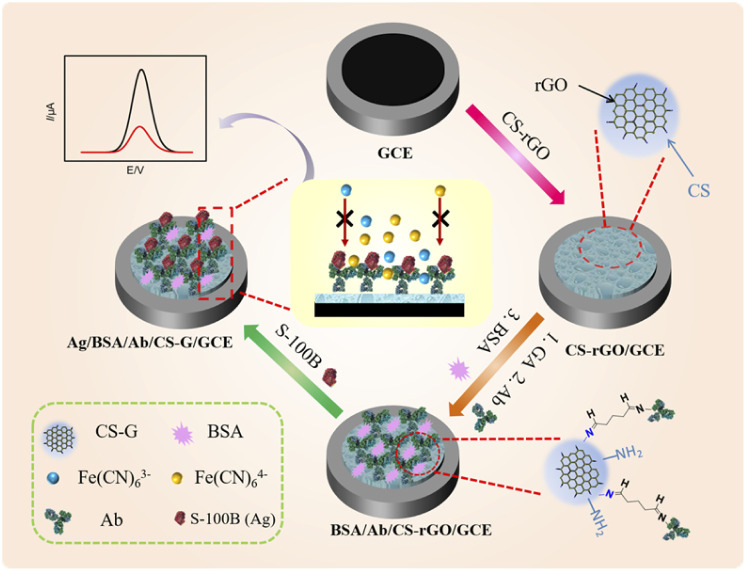
Schematic illustration for the fabrication of immunosensor based on CS–rGO and the electrochemical determination of S-100B.

Although graphene has excellent electron transfer rate and high specific surface area, it has highly hydrophobic surface and strong van der Waals force between layers. Thus, graphene is commonly difficult to be dispersed in water and the agglomeration caused by interlayer stacking will significantly reduce its specific surface area. However, the precursor for the preparation of rGO, GO, is hydrophilic and has good dispersibility in water.^34^ As illustrated in Fig. 1, CS–rGO with good dispersibility in aqueous solution was prepared in this work. Briefly, GO was reduced by hydrazine in the presence of CS. The CS–rGO nanocomposite drop-coated on GCE provided abundant amine groups to react with GA to introduce aldehyde groups for covalent immobilization of recognitive Ab. After blocking the nonspecific site with BSA, the immunosensor was prepared.

**Fig. 2:**
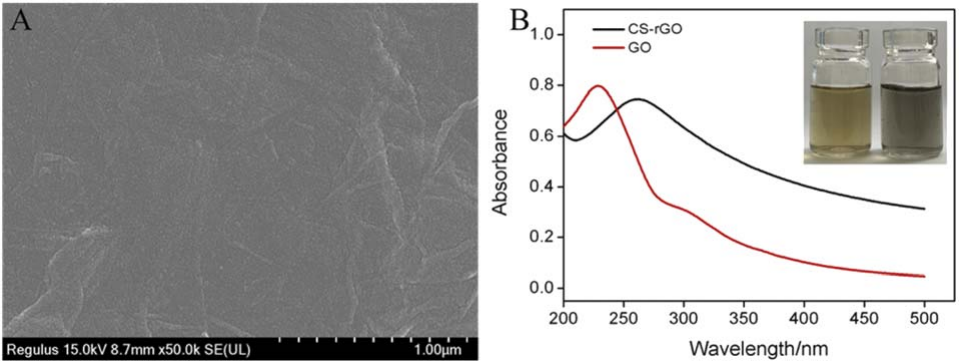
(A) SEM image of CS–rGO film modified on GCE. (B) UV-vis spectra of GO and CS–rGO.

The morphology of the synthesized CS–rGO was characterized by SEM. As shown in Fig. 2A, CS–rGO has a unique folded structure of graphene. TEM images of GO and CS–rGO were also given in Fig. S1.† As shown, GO exhibited a thin-layered structure with some wrinkles. After reduction by hydrazine and binding to CS, the as-prepared CS–rGO nanocomposite retained the characteristic sheet-like structure. In addition, no agglomerated structure was observed, indicating that the reduction of GO in the presence of CS can avoid agglomeration of graphene material. The morphology and thickness of CS–rGO were further verified by AFM. As shown in Fig. S2,† CS–rGO was single-layered sheet with a thickness of about 3.5 nm. This thickness is higher than that of monolayer rGO (commonly around 1.0 nm). As CS bound on the surface of rGO through electrostatic or hydrogen bonding or van der Waals forces, the thickness of CS–rGO increased. Thus, the composite of chitosan with rGO did not change the monolayer structure of rGO, but resulted in an increase in its sheet thickness.

The successful composite of CS and rGO was characterized by ultraviolet-visible absorption spectroscopy (UV-vis, Fig. 2B). In case of GO, the π–π* transitions of C=C and the n–π* transitions of C=O were revealed corresponding to 230 nm and 300 nm, respectively. When GO reacted with hydrazine, on the one hand, the π–π* transitions of C=C red-shifted to 265 nm, proving that the increase of the conjugated structure through the reduction of GO. In addition, the absorption peak corresponding to the n–π* transition became indistinct,^35^ which was attributed to the reduction of surface oxygen-containing functional groups after GO is reduced to rGO. These phenomena demonstrated the successful preparation of CS–rGO.

**Fig. 3:**
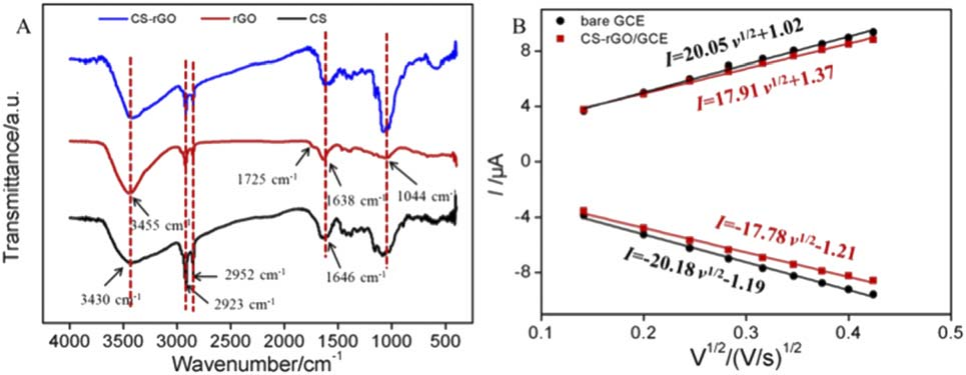
(A) FR-IR spectra of CS, CS–rGO, and rGO synthesized without CS. (B) Relationship between *I vs. v*^1/2^ derived from the CVs of bare GCE and CS–rGO/GCE obtained in 0.5 mM K_3_[Fe(CN)_6_] containing 0.1 M KCl.

Fig. 3A showed the FR-IR spectra of three materials including CS, rGO, and CS–rGO. Amongst, rGO was obtained by reduction of GO under the same conditions without CS. For chitosan, the peaks at 3430 cm^−1^ corresponded to the stretching vibration absorption peak of –OH, peaks at 2923 cm^−1^ and 2852 cm^−1^ were the stretching vibration absorption peaks of C–H. The presence of the characteristic peak of N–H at 1646 cm^−1^ indicated that there had chemically reactive amino groups in chitosan.^36,37^ For rGO, the vibrational absorption peak of –OH at 3455 cm^−1^, the stretching vibration of C=O at 1725 cm^−1^, and the stretching vibration peak of C=C at 1638 cm^−1^, and the stretching vibration peak of C–O–C at 1044 cm^−1^ revealed both the sp^2^ carbon structure and the surface oxygen-containing groups on rGO.^38^ In case of CS–rGO, the FT-IR spectrum contained characteristic absorption peaks of rGO and CS, indicating successful preparation of the nanocomposite material.

The changes of chemical groups during the preparation of CS–rGO from GO were further investigated by XPS. As shown in Fig. S3A and B,† the high-resolution C 1s spectra of both GO and CS–G displayed four types of carbon atoms, including C–C/C=C (sp^2^ carbon atoms, 284.6 eV), C–O (epoxy or alkoxy group, 286.7 eV), C=O (aldehyde or ketone group, 287.2 eV) and O–C=O (carboxyl group, 288.5 eV). However, the C–/C=C peak in CS–rGO became larger, and the peak intensities of C=O and COOH greatly reduced, indicating that GO was successfully reduced. In the N 1s spectrum of CS–rGO in Fig. S3C,† characteristic peaks of –NH_2_/–NH– and –NH^3+^ appeared, proving that chitosan successfully composited on rGO.

Fig. S4† displayed the Raman spectra of GO and CS–rGO. As shown, two prominent peaks at ∼1350 cm^−1^ (D band) and ∼1590 cm^−1^ (G band) were observed, which corresponded to a breathing mode of *κ*-point photons of A_1g_ symmetry and the first order scattering of the E_2g_ phonon of sp^2^ C atoms, respectively. When GO was reduced in presence of CS, the obtained CS–rGO nanocomposite had a higher intensity ratio (*r* = *I*_D_/*I*_G_, 1.32) in comparison with that of GO (*r* = 0.85), suggesting the increase of sp^3^ defects within the sp^2^ carbon network upon the reduction.^39^

### 3.2 Electrochemical characterization of CS–rGO/ITO

Fig. S5A† showed the cyclic voltammetry (CV) curves of CS/GCE and CS–rGO/GCE in Fe(CN)_6_^3−/4−^ solution. As seen, CS–rGO/GCE had a significantly improved electrochemical signal compared with that of CS/GCE, which was attributed to the high electron transfer rate of rGO. It was noteworthy that the peak current obtained on CS–rGO/GCE exhibited good stability during the continuous 20 scanning cycles (Fig. S5B†). The peak current remained 98% of the initial signal after 20 consecutive scans. In contrast, the electrochemical signal of redox probe remarkably increased when CS/GCE was continuously scanned (Fig. S6†), indicating the instability of the modified CS layer resulting from the swelling or shedding of the CS. Thus, the introduced rGO can significantly improve the stability of CS film, which might be ascribed to the cross-linking between CS and rGO through electrostatic interaction or hydrogen bonding or van der Waals force.

The effect of scan rate on the electrochemical response was also investigated. Fig. S7† showed the cyclic voltammetry curves obtained on CS–rGO/GCE or bare GCE at different scan rates. As seen from Fig. 3B, the peak current had a good linear relationship with the square root of the scan rate, proving a diffusion-controlled process on both electrodes. The electrochemical active surface area (ECSA) of bare GCE can be calculated to be 0.058 cm^2^ by the Randles–Sevcik equation,^40–42^ while that of CS–rGO/GCE was 0.052 cm^2^.

### 3.3 Fabrication of immunosensor

**Fig. 4:**
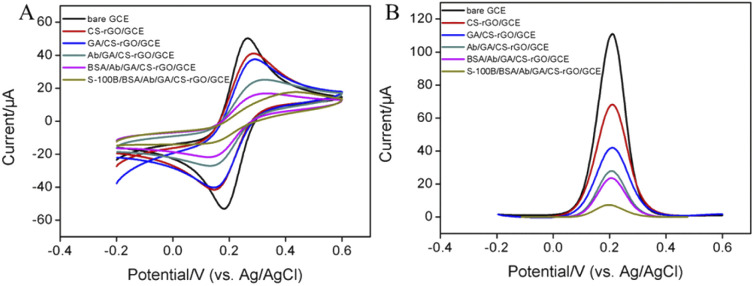
CV (A) and DPV (B) curves obtained on different electrodes obtained in the fabrication of the immunosensor or after incubated with S-100B in Fe(CN)_6_^3−/4−^ solution.

Fig. 4A displayed the CV curves of Fe(CN)_6_^3−/4−^ on different electrodes obtained during the construction of the immunosensor. When CS–rGO was modified on the surface of GCE, the CV peak signal of the electrochemical probe on the CS–rGO/GCE electrode was weakly reduced accompanying a slightly larger peak-to-peak difference in comparison with GCE since CS was a non-conductive biopolymer.^43^ After further activation by GA, the peak signal on GA/CS–rGO/GCE slightly reduced due to the possible cross-linking between CS molecules. The following covalent immobilization of Ab (Ab/GA/CS–rGO/GCE) and BSA blocking (BSA/Ab/GA/CS–rGO/GCE) resulted in a significant decrease in the current signal and a further increase in the peak-to-peak difference, which was attributed to the hindered electron transfer by the non-conductive protein layer. When the fabricated immunosensor (BSA/Ab/GA/CS–rGO/GCE) was incubated with S-100B, only extremely weak probe signal was detected. Thus, the formation of antigen–antibody complexes significantly reduced electron transfer on the electrode surface. The DPV curves in Fig. 4B demonstrated the consistent results, indicating efficient fabrication of the immunosensor and the specific recognition of S-100B on the immuno-recognitive surface.

### 3.4 Electrochemical determination of S-100B using the developed immunosensor

**Fig. 5:**
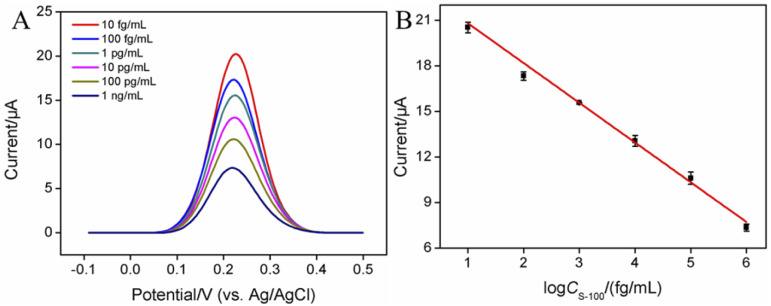
(A) DPV curves of the immunosensor in the presence of different concentration (10 fg mL^−1^ to 1 ng mL^−1^) of S-100B. (B) Calibration curve for the determination of S-100B.

The formation of antigen–antibody complex on the surface of immunosensor significantly reduced the signal of the redox probe (Fe(CN)_6_^3−^/Fe(CN)_6_^4−^), realizing electrochemical determination of S-100B. The performance of the developed immunosensor for the electrochemical determination of S-100B was investigated. Fig. 5A displayed DPV curves obtained on the fabricated immunosensor, BSA/Ab/GA/CS–rGO/GCE, in presence of different concentration of S-100B. As shown, the peak current gradually decreased when the concentration of S-100B increased. A linear correlation was revealed between the peak current (*I*, μA) and the logarithm value of the concentration of S-100B (log *C*) in the range from 10 fg mL^−1^ to 1 ng mL^−1^ (*I* = −2.62 log *C*_S-100B_ + 23.4, *R*^2^ = 0.990, Fig. 5B). The limit of detection (LOD) was 1.9 fg mL^−1^ at a signal-to-noise ratio of 3 (S/N = 3).

Comparison between determination of S-100B using different methods was demonstrated in Table S1.†^44–49^ The LOD is lower than that obtained using fluorescent determination based on antibody-conjugated magnetic beads and quantum dots (MB-Ab/S100B/Ab-QD),^44^ or electrochemical impedance spectroscopy (EIS) determination based on antibody binding to cysteamine modified on interdigitate-zigzag biochip using glutaraldehyde as cross-linking agent (IDZB/Cys/GA/anti-S100B),^45^ electrochemical determination based on Au-coated magnetic NPs/thiol-ended Ab,^46^ but higher than photoelectrochemical determination based on immobilization of Ab on green reduced graphene oxide and decorated with gold nanoparticles (Ab/rGO–Au),^47^ or electrochemical determination by immobilization of Ab on 4-nitrobenzenediazonium/GA modified graphene screen printed electrode (GSPE/4-NBD/GA/Ab),^48^ or poly(ethyleneimine) modified poly(methyl methacrylate) (PEI–PMMA) modified electrode.^49^

### 3.5 Selectivity of the immunosensor

**Fig. 6:**
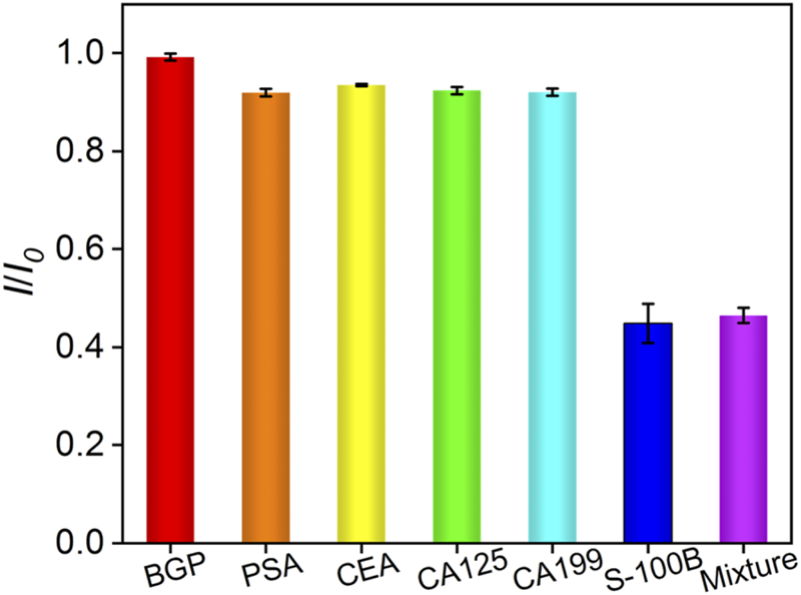
The currents obtained on the developed immunosensor in absence (control) or presence of the indicated tumor biomarker.

To further explore the selectivity of the immunosensor, the response obtained when the immunosensor was incubated with several common tumor biomarkers including BGP, PSA, CEA, CA125, CA199, were investigated. As shown in Fig. 6, the current obtained on the immunosensor almost did not change in absence (control) or presence of one of the possible interferences. On the contrary, the peak current decreased greatly when S-100B or the mixture of all proteins was added.

### 3.6 Reproducibility and stability of the immunosensor

The reproducibility and stability of the constructed immunosensor were further investigated. Five immunosensors were prepared in parallel. After the fabricated immunosensors were incubated with S-100B (0.1 ng mL^−1^), the current signal of the electrochemical probe on different electrodes were similar with a low relative standard deviation (RSD, 1.8%), indicating that the electrode fabrication had good reproducibility. The storage stability of the developed immunosensor was also investigated. When the immunosensor was stored at 4 °C for different times, the RSD for the determination of S-100B (10 pg mL^−1^) was 2.1%, demonstrating high stability.

### 3.7 Determination of S-100B in human serum

Electrochemical determination of S-100B in human serum was investigated using standard addition method. Briefly, different concentrations of S-100B were artificially added into the human serum. Then, the human serum was diluted by a factor of 50. As shown, the recovery of the concentration of S-100B ranged from 97.4% to 105% with an RSD no more than 3.5% (Table 1), indicating good reliability for the determination of S-100B.

**Table 1:** Determination of S-100B in human serum samples

Sample	Added (pg mL^−1^)	Found (pg mL^−1^)	RSD (%, *n* = 3)	Recovery (%)
Human serum	5.00	4.87	3.4	97.4
	50.0	52.6	1.4	105
	500	488	3.5	97.6

## 4. Conclusions

In summary, a simple immunosensor sensor was developed for sensitive determination of S-100B based on the modification of electrode using chitosan–reduced graphene oxide nanocomposite. CS–rGO nanocomposites integrate the functional properties of graphene and chitosan, exhibiting two-dimensional monolayer structure, good electrical conductivity, biocompatibility, and modifiability. In comparison with pure CS film, the interaction between CS and rGO improve the stability of the film. The amino groups in CS molecules were activated by the functional linker, glutaraldehyde, for the further covalent immobilization of the recognitive antibody. As the formation of the antigen–antibody complex decreases the signal of the redox probe, the electrochemical determination of S-100B was realized with wide dynamic range from 10 fg mL^−1^ to 1 ng mL^−1^ and a low limit of detection of 1.9 fg mL^−1^. Owing to the biocompatible environment for the immobilization of Ab and the good electron transfer of rGO, the developed sensor has advantages of high sensitivity and good stability, indicating great potential for the determination of tumor biomarkers.

## Ethical statement

Human serum was used in this work. Informed consent was obtained from all human subjects.

## Conflicts of interest

There are no conflicts to declare.

## Supplementary Material

RA-012-D2RA04208H-s001

## References

[cit1] Davis L. E., Shalin S. C., Tackett A. J. (2019). Current state of melanoma diagnosis and treatment. Cancer Biol. Ther..

[cit2] Saglam O., Naqvi S. M. H., Zhang Y., Mesa T., Teer J. K., Yoder S., Lee J., Messina J. (2018). Female genitourinary tract melanoma: mutation analysis with clinicopathologic correlation, a single-institution experience. Melanoma Res..

[cit3] Abraha H., Fuller L., Du Vivier A., Higgins E., Sherwood R. (1997). Serum S-100 protein: a potentially useful prognostic marker in cutaneous melanoma. Br. J. Dermatol..

[cit4] Goldstein A. M., Stidd K. C., Yang X. R., Fraser M. C., Tucker M. A. (2018). Pediatric melanoma in melanoma-prone families. Cancer.

[cit5] Quintanilla-Dieck M. J., Bichakjian C. K. (2019). Management of early-stage melanoma. Facial Plast. Surg. Clin..

[cit6] Gebhardt C., Lichtenberger R., Utikal J. (2016). Biomarker value and pitfalls of serum S100B in the follow-up of high-risk melanoma patients. J. Dtsch. Dermatol. Ges..

[cit7] Korfias S., Stranjalis G., Boviatsis E., Psachoulia C., Jullien G., Gregson B., Mendelow A. D., Sakas D. E. (2007). Serum S-100B protein monitoring in patients with severe traumatic brain injury. Intensive Care Med..

[cit8] Ettinger A., Laumark A. B., Ostroff R. M., Brundell J., Baumgartner W. A., Razumovsky A. Y. (1999). A new optical immunoassay for detection of S-100B protein in whole blood. Ann. Thorac. Surg..

[cit9] Green A. J., Keir G., Thompson E. (1997). A specific and sensitive ELISA for measuring S-100b in cerebrospinal fluid. J. Immunol. Methods.

[cit10] Bonfrer J., Korse C., Nieweg O., Rankin E. (1998). The luminescence immunoassay S-100: a sensitive test to measure circulating S-100B: its prognostic value in malignant melanoma. Br. J. Cancer.

[cit11] Mussack T., Klauss V., Ruppert V., Gippner-Steppert C., Biberthaler P., Schiemann U., Hoffmann U., Jochum M. (2006). Rapid measurement of S-100B serum protein levels by Elecsys S100 immunoassay in patients undergoing carotid artery stenting or endarterectomy. Clin. Biochem..

[cit12] Gong J., Zhang T., Luo T., Luo X., Yan F., Tang W., Liu J. (2022). Bipolar silica nanochannel array confined electrochemiluminescence for ultrasensitive detection of SARS-CoV-2 antibody. Biosens. Bioelectron..

[cit13] Ma K., Zheng Y., An L., Liu J. (2022). Ultrasensitive Immunosensor for Prostate-Specific Antigen Based on Enhanced Electrochemiluminescence by Vertically Ordered Mesoporous Silica-Nanochannel Film. Front. Chem..

[cit14] Lin J., Li K., Wang M., Chen X., Liu J., Tang H. (2020). Reagentless and sensitive determination of carcinoembryonic antigen based on a stable Prussian blue modified electrode. RSC Adv..

[cit15] Gong J., Zhang T., Chen P., Yan F., Liu J. (2022). Bipolar silica nanochannel array for dual-mode electrochemiluminescence and electrochemical immunosensing platform. Sens. Actuators, B.

[cit16] Zhang J., Yang L., Pei J., Tian Y., Liu J. (2022). A reagentless electrochemical immunosensor for sensitive detection of carcinoembryonic antigen based on the interface with redox probe-modified electron transfer wires and effectively immobilized antibody. Front. Chem..

[cit17] Yan Y., Gong J., Chen J., Zeng Z., Huang W., Pu K., Liu J., Chen P. (2019). Recent advances on graphene quantum dots: From chemistry and physics to applications. Adv. Mater..

[cit18] Liu X., Chen Z., Wang T., Jiang X., Qu X., Duan W., Xi F., He Z., Wu J. (2022). Tissue imprinting on 2D nanoflakes-capped silicon nanowires for lipidomic mass spectrometry imaging and cancer diagnosis. ACS Nano.

[cit19] Xi F., Zhao J., Shen C., He J., Chen J., Yan Y., Li K., Liu J., Chen P. (2019). Amphiphilic graphene quantum dots as a new class of surfactants. Carbon.

[cit20] Zhou H., Ding Y., Su R., Lu D., Tang H., Xi F. (2022). Silica nanochannel array film supported by β-cyclodextrin-functionalized graphene modified gold film electrode for sensitive and direct electroanalysis of acetaminophen. Front. Chem..

[cit21] Yan F., Luo T., Jin Q., Zhou H., Sailjoi A., Dong G., Liu J., Tang W. (2021). Tailoring molecular permeability of vertically-ordered mesoporous silica-nanochannel films on graphene for selectively enhanced determination of dihydroxybenzene isomers in environmental water samples. J. Hazard. Mater..

[cit22] Yan F., Chen J., Jin Q., Zhou H., Sailjoi A., Liu J., Tang W. (2020). Fast one-step fabrication of a vertically-ordered mesoporous silica-nanochannel film on graphene for direct and sensitive detection of doxorubicin in human whole blood. J. Mater. Chem. C.

[cit23] Gong J., Tang H., Wang M., Lin X., Wang K., Liu J. (2022). Novel three-dimensional graphene nanomesh prepared by facile electro-etching for improved electroanalytical performance for small biomolecules. Mater. Des..

[cit24] Gong J., Tang H., Luo X., Zhou H., Lin X., Wang K., Fei Y., Xi F., Liu J. (2021). Vertically ordered mesoporous silica-nanochannel film-equipped three-dimensional macroporous graphene as sensitive electrochemiluminescence platform. Front. Chem..

[cit25] Zhu X., Xuan L., Gong J., Liu J., Wang X., Xi F., Chen J. (2022). Three-dimensional macroscopic graphene supported vertically-ordered mesoporous silica-nanochannel film for direct and ultrasensitive detection of uric acid in serum. Talanta.

[cit26] Lu Z., Wu L., Dai X., Wang Y., Sun M., Zhou C., Du H., Rao H. (2021). Novel flexible bifunctional amperometric biosensor based on laser engraved porous graphene array electrodes: Highly sensitive electrochemical determination of hydrogen peroxide and glucose. J. Hazard. Mater..

[cit27] Ma X., Liao W., Zhou H., Tong Y., Yan F., Tang H., Liu J. (2020). Highly sensitive detection of rutin in pharmaceuticals and human serum using ITO electrodes modified with vertically-ordered mesoporous silica–graphene nanocomposite films. J. Mater. Chem. B.

[cit28] Zhou H., Ma X., Sailjoi A., Zou Y., Lin X., Yan F., Su B., Liu J. (2022). Vertical silica nanochannels supported by nanocarbon composite for simultaneous detection of serotonin and melatonin in biological fluids. Sens. Actuators, B.

[cit29] Duan W., Jin Y., Cui Y., Xi F., Liu X., Wo F., Wu J. (2021). A co-delivery platform for synergistic promotion of angiogenesis based on biodegradable, therapeutic and self-reporting luminescent porous silicon microparticles. Biomaterials.

[cit30] Cui Y., Duan W., Jin Y., Wo F., Xi F., Wu J. (2021). Graphene quantum dot-decorated luminescent porous silicon dressing for theranostics of diabetic wounds. Acta Biomater..

[cit31] Cui Y., Duan W., Jin Y., Wo F., Xi F., Wu J. (2020). Ratiometric fluorescent nanohybrid for noninvasive and visual monitoring of sweat glucose. ACS Sens..

[cit32] Jirakunakorn R., Khumngern S., Choosang J., Thavarungkul P., Kanatharana P., Numnuam A. (2020). Uric acid enzyme biosensor based on a screen-printed electrode coated with Prussian blue and modified with chitosan-graphene composite cryogel. Microchem. J..

[cit33] Prajapati D., Pal A., Dimkpa C., Singh U., Devi K. A., Choudhary J. L., Saharan V. (2022). Chitosan nanomaterials: A prelim of next-generation fertilizers; existing and future prospects. Carbohydr. Polym..

[cit34] Liu J., Guo S., Han L., Ren W., Liu Y., Wang E. (2012). Multiple pH-responsive graphene composites by non-covalent modification with chitosan. Talanta.

[cit35] Wang C., Chen B., Zou M., Cheng G. (2014). Cyclic RGD-modified chitosan/graphene oxide polymers for drug delivery and cellular imaging. Colloids Surf., B.

[cit36] Li X., Han Y., Ling Y., Wang X., Sun R. (2015). Assembly of layered silicate loaded quaternized chitosan/reduced graphene oxide composites as efficient absorbents for double-stranded DNA. ACS Sustainable Chem. Eng..

[cit37] Li D., Muller M. B., Gilje S., Kaner R. B., Wallace G. G. (2008). Processable aqueous dispersions of graphene nanosheets. Nat. Nanotechnol..

[cit38] Mianehrow H., Afshari R., Mazinani S., Sharif F., Abdouss M. (2016). Introducing a highly dispersed reduced graphene oxide nano-biohybrid employing chitosan/hydroxyethyl cellulose for controlled drug delivery. Int. J. Pharm..

[cit39] Kumar S., Koh J. (2014). Physiochemical and optical properties of chitosan based graphene oxide bionanocomposite. Int. J. Biol. Macromol..

[cit40] Ashraf G., Asif M., Aziz A., Iftikhar T., Zhong Z. T., Zhang S., Liu B., Chen W., Zhao Y. D. (2022). Advancing interfacial properties of carbon cloth via anodic-induced self-assembly of MOFs film integrated with alpha-MnO2: a sustainable electrocatalyst sensing acetylcholine. J. Hazard. Mater..

[cit41] Alam A. U., Deen M. J. (2020). Bisphenol A electrochemical sensor using graphene oxide and beta-cyclodextrin-functionalized multi-walled carbon nanotubes. Anal. Chem..

[cit42] Elgrishi N., Rountree K. J., McCarthy B. D., Rountree E. S., Eisenhart T. T., Dempsey J. L. (2017). A practical beginner's guide to cyclic voltammetry. J. Chem. Educ..

[cit43] Afkhami A., Hashemi P., Bagheri H., Salimian J., Ahmadi A., Madrakian T. (2017). Impedimetric immunosensor for the label-free and direct detection of botulinum neurotoxin serotype A using Au nanoparticles/graphene-chitosan composite. Biosens. Bioelectron..

[cit44] Kim C., Searson P. C. (2015). Magnetic bead-quantum dot assay for detection of a biomarker for traumatic brain injury. Nanoscale.

[cit45] Kuo Y. C., Lee C. K., Lin C. T. (2018). Improving sensitivity of a miniaturized label-free electrochemical biosensor using zigzag electrodes. Biosens. Bioelectron..

[cit46] Hassanain W. A., Sivanesan A., Ayoko G. A., Izake E. L. (2020). Rapid electrochemical nanosensing of S100β in blood. J. Electrochem. Soc..

[cit47] Tabrizi M. A., Ferre-Borrull J., Kapruwan P., Marsal L. F. (2019). A photoelectrochemical sandwich immunoassay for protein S100beta, a biomarker for Alzheimer's disease, using an ITO electrode modified with a reduced graphene oxide-gold conjugate and CdS-labeled secondary antibody. Microchim. Acta.

[cit48] Khetani S., Aburashed R., Singh A., Sen A., Sanati-Nezhad A. (2017). Immunosensing of S100β biomarker for diagnosis of spinal cord injuries (SCI). Sens. Actuators, B.

[cit49] Liu Y., Wang H., Chen J., Liu C., Li W., Kong J., Yang P., Liu B. (2013). A sensitive microchip-based immunosensor for electrochemical detection of low-level biomarker S100B. Electroanalysis.

