# Supplementary material for: A simple electrochemical immunosensor based on a chitosan/reduced graphene oxide nanocomposite for sensitive detection of biomarkers of malignant melanoma
Source: RSC Adv. 2022 Sep 12;12(40):25844–51. doi: 10.1039/d2ra04208h (PMC9465697; doi:10.1039/d2ra04208h)
Supplement: RA-012-D2RA04208H-s001 [file RA-012-D2RA04208H-s001.pdf]

Electronic Supplementary Material (ESI) for RSC Advances.
This journal is © The Royal Society of Chemistry 2022

TABLES

Table S1. Comparison between detection of S-100B using different method.

Materials	Method	Liner range (ng/mL)	LOD (pg/mL)	Ref.
MB-Ab/S100B/Ab-QD	Fluorescence	0.01-30	10	44
IDZB/Cys/GA/anti-S100B	EIS	$10\text{-}10^4$	10^4	45
Au-coated magnetic NPs/ thiol-ended Ab	DPV	3.7-37	3.7×10^3	46
Ab/rGO-Au	Photoelectrochemical	$2.5\times10^{-4}\text{-}1$	0.15	47
GSPE/4-NBD/GA/Ab	DPV	1-10	1	48
PEI-PMMA/Ab	DPV	$10^{-4}\text{-}10^{-1}$	0.1	49
Ab/GA/CS-rGO/GCE	DPV	$10^{-2}\text{-}10^3$	1.9	Our work

MB: magnetic beads; QD: quantum dots; IDZB: interdigitate-zigzag biochip; Cys: cysteamine; EIS: electrochemical impedance spectroscopy; NPs: nanoparticles; rGO-Au: green reduced graphene oxide and decorated with gold nanoparticles; GSPE: graphene screen printed electrodes; 4-NBD: 4-nitrobenenediazonium; GA: glutaraldehyde; PEI-PMMA: poly(ethyleneimine) modified poly(methyl methacrylate).

FIGURES

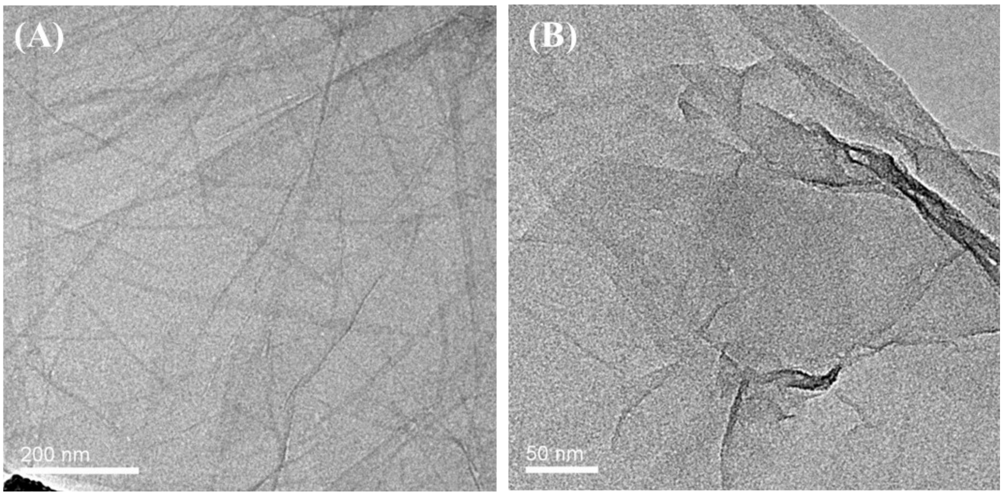

Fig. S1 TEM images of GO (A) and CS-rGO (B).

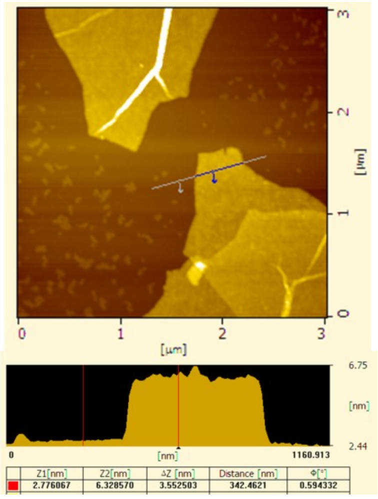

Fig. S2 AFM image (upper) and the corresponding height profiles (below) along the indicated line of CS-rGO.

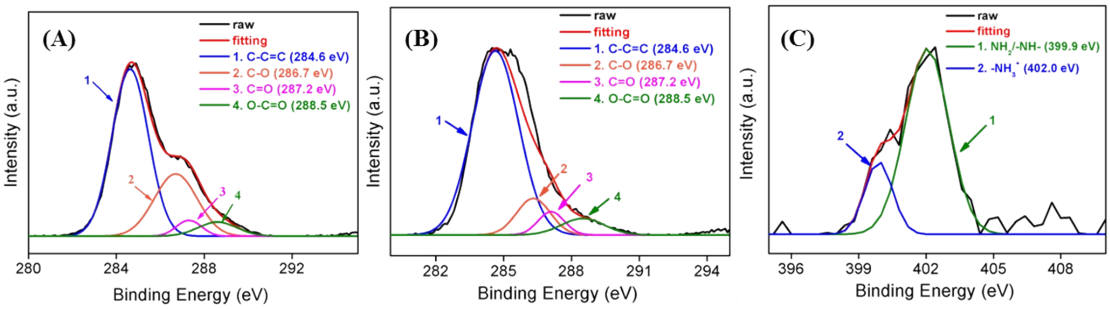

Fig. S3 High-resolution C1s XPS profiles of GO (A) and CS-G (B). High-resolution N1s XPS profile of CS-G (C).

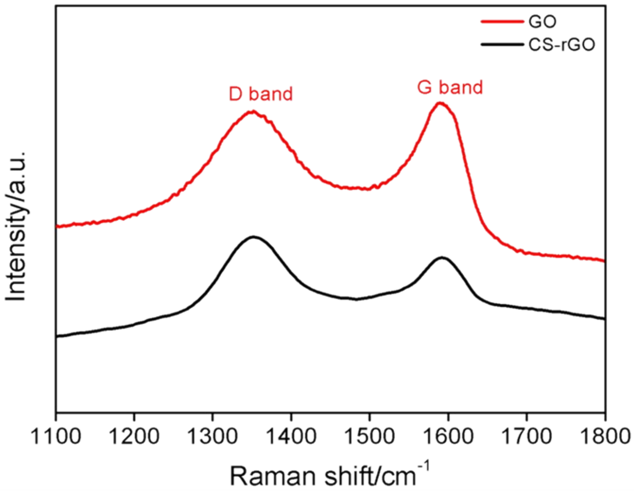

Fig. S4 Raman spectra of GO and CS-rGO.

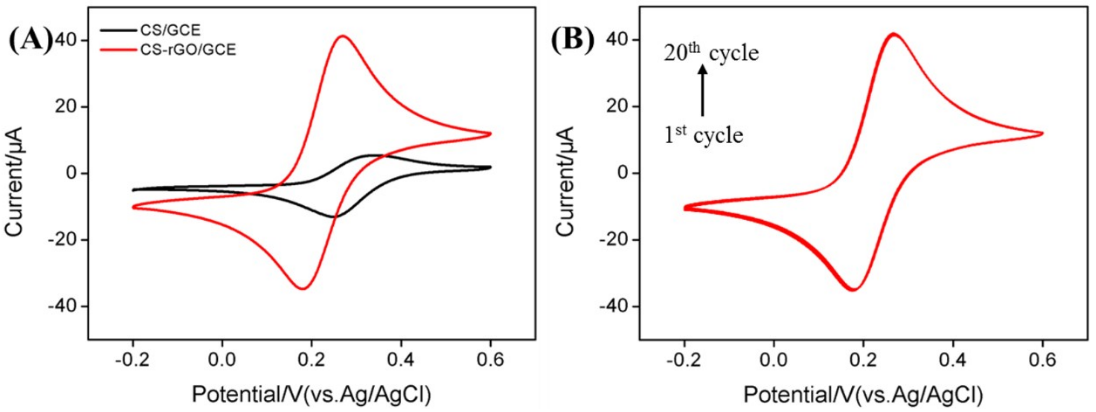

Fig. S5 (A) CV curves obtained on CS/GCE and CS-rGO/GCE. (B) CV curves on CS-rGO/GCE during the continuous 20 scanning cycles. The electrolyte is $Fe(CN)_6^{3-/4-}$ (2.5 mM) containing KCl (0.1 M).

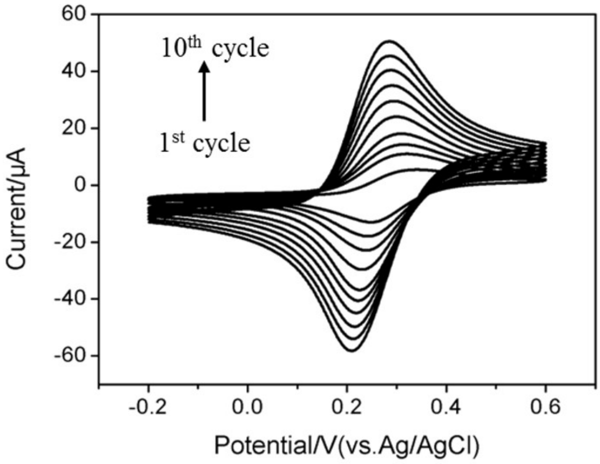

Fig. S6 CV curves on CS/GCE during the continuous 10 scanning cycles in $Fe(CN)_6^{3-/4-}$ (2.5 mM) containing KCl (0.1 M).

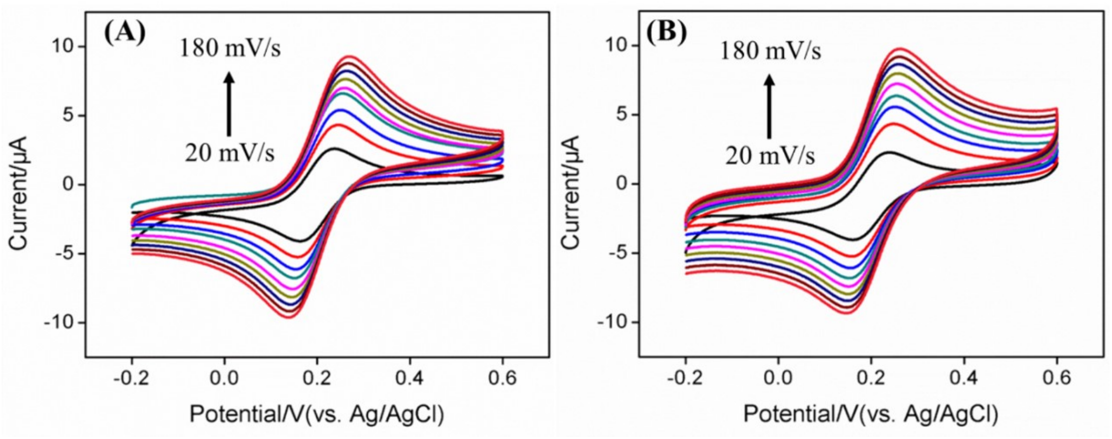

Fig. S7 CV curves of bare GCE (A) CS-rGO/GCE (B) at different scan rates in 0.1 M KCl containing 0.5 mM $Fe(CN)_6^{3-/4-}$.
